# 

**Published:** 1848-03

**Authors:** 


### CONTENTS

OF NO. III.

### ORIGINAL COMMUNICATIONS.

ESSAYS AND CASES.

ART. I.—Statistical Medicine or Numerical Analysis applied to the Investigation of Morbid Actions: A Lecture, delivered, by request, to the Medical Class of the University of Louisville, January 17, 1848. By SAMUEL A. CARTWRIGHT, M.D., of Natchez, Miss. .... 185

ART. II.—A brief Account of a Fever which prevailed in the neighborhood of Charlotte, Tennessee, during the Summer of 1846. By JAS. M. LARKINS, M.D., of Charlotte, Tennessee .... 206

ART. III.—Notes on Medical Matters and Medical Men in Paris. By DAVID W. YANDELL, M.D., of Louisville, Kentucky .... 209

REVIEWS.

ART. IV.—Etherization: with Surgical Remarks. By JOHN C. WARREN, M. D., Emeritus Professor of Anatomy and Surgery in the University at Cambridge; Surgeon at Massachusetts General Hospital; Honorary Member of the Medical and Chirurgical Society of London; Corresponding Member of the Royal Academy of Medicine at Paris, and of the Academies of Naples, Florence, etc., etc. .... 233

ART. V.—An Attempt to discover some of the Laws which govern Animal Torpidity and Hibernation. By PETER A. BROWNE, LL.D., Member of the National Institute for the Promotion of Science, of the American Association for the Promotion of Science, of the Academy of Natural Science of Philadelphia, etc., etc. Read before the Association of American Geologists and Naturalists, at the eighth Annual Meeting, in Boston, in September, 1847 .... 240

SELECTIONS FROM AMERICAN AND FOREIGN JOURNALS.

On the Advance of the Asiatic Cholera; with suggestions for its Treatment.........245
On Etherization in Labor.........254
Permanent Retention of Antimony in the living Organs.........258
Rape under the influence of Ether.........263
The nature of General Shields' Wound.........264
Chloroform.........264
Stomatitis Materna.—The Sore Mouth of Nursing Women.........266

ORIGINAL INTELLIGENCE.

Chloroform.........269
Physiological Temperance Society of the University of Louisville..273
Deaths in the Medical Class.........273
Ship Typhus Fever in New Orleans.........274
Second Medical School in Louisville.........275
Extension of the Medical Session in our University.........276

